# Economics and Equity of Large Language Models: Health Care Perspective

**DOI:** 10.2196/64226

**Published:** 2024-11-14

**Authors:** Radha Nagarajan, Midori Kondo, Franz Salas, Emre Sezgin, Yuan Yao, Vanessa Klotzman, Sandip A Godambe, Naqi Khan, Alfonso Limon, Graham Stephenson, Sharief Taraman, Nephi Walton, Louis Ehwerhemuepha, Jay Pandit, Deepti Pandita, Michael Weiss, Charles Golden, Adam Gold, John Henderson, Angela Shippy, Leo Anthony Celi, William R Hogan, Eric K Oermann, Terence Sanger, Steven Martel

**Affiliations:** 1 Children's Hospital of Orange County Orange, CA United States; 2 Fred Hutch Patient Care Seattle, WA United States; 3 Amazon Web Services Detroit, MI United States; 4 Nationwide Children's Hospital Columbus, OH United States; 5 Amazon Web Services San Francisco, CA United States; 6 University of California Irvine Irvine, CA United States; 7 Children’s Hospital of Orange County Orange, CA United States; 8 Amazon Web Services Seattle, WA United States; 9 University of California Irvine Health Irvine, CA United States; 10 Cognoa LLC Palo Alto, CA United States; 11 National Institutes of Health Bethesda, MD United States; 12 Scripps Research Translational Institute La Jolla, CA United States; 13 Amazon Web Services Houston, TX United States; 14 Massachusetts Institute of Technology Cambridge, MA United States; 15 Medical College of Wisconsin Milwaukee, WI United States; 16 NYU Langone Medical Center New York, NY United States; 17 Physicians Specialty Faculty Orange, CA United States

**Keywords:** large language model, LLM, health care, economics, equity, cloud service providers, cloud, health outcome, implementation, democratization

## Abstract

Large language models (LLMs) continue to exhibit noteworthy capabilities across a spectrum of areas, including emerging proficiencies across the health care continuum. Successful LLM implementation and adoption depend on digital readiness, modern infrastructure, a trained workforce, privacy, and an ethical regulatory landscape. These factors can vary significantly across health care ecosystems, dictating the choice of a particular LLM implementation pathway. This perspective discusses 3 LLM implementation pathways—training from scratch pathway (TSP), fine-tuned pathway (FTP), and out-of-the-box pathway (OBP)—as potential onboarding points for health systems while facilitating equitable adoption. The choice of a particular pathway is governed by needs as well as affordability. Therefore, the risks, benefits, and economics of these pathways across 4 major cloud service providers (Amazon, Microsoft, Google, and Oracle) are presented. While cost comparisons, such as on-demand and spot pricing across the cloud service providers for the 3 pathways, are presented for completeness, the usefulness of managed services and cloud enterprise tools is elucidated. Managed services can complement the traditional workforce and expertise, while enterprise tools, such as federated learning, can overcome sample size challenges when implementing LLMs using health care data. Of the 3 pathways, TSP is expected to be the most resource-intensive regarding infrastructure and workforce while providing maximum customization, enhanced transparency, and performance. Because TSP trains the LLM using enterprise health care data, it is expected to harness the digital signatures of the population served by the health care system with the potential to impact outcomes. The use of pretrained models in FTP is a limitation. It may impact its performance because the training data used in the pretrained model may have hidden bias and may not necessarily be health care–related. However, FTP provides a balance between customization, cost, and performance. While OBP can be rapidly deployed, it provides minimal customization and transparency without guaranteeing long-term availability. OBP may also present challenges in interfacing seamlessly with downstream applications in health care settings with variations in pricing and use over time. Lack of customization in OBP can significantly limit its ability to impact outcomes. Finally, potential applications of LLMs in health care, including conversational artificial intelligence, chatbots, summarization, and machine translation, are highlighted. While the 3 implementation pathways discussed in this perspective have the potential to facilitate equitable adoption and democratization of LLMs, transitions between them may be necessary as the needs of health systems evolve. Understanding the economics and trade-offs of these onboarding pathways can guide their strategic adoption and demonstrate value while impacting health care outcomes favorably.

## Introduction

### Overview

**Figure 1:**
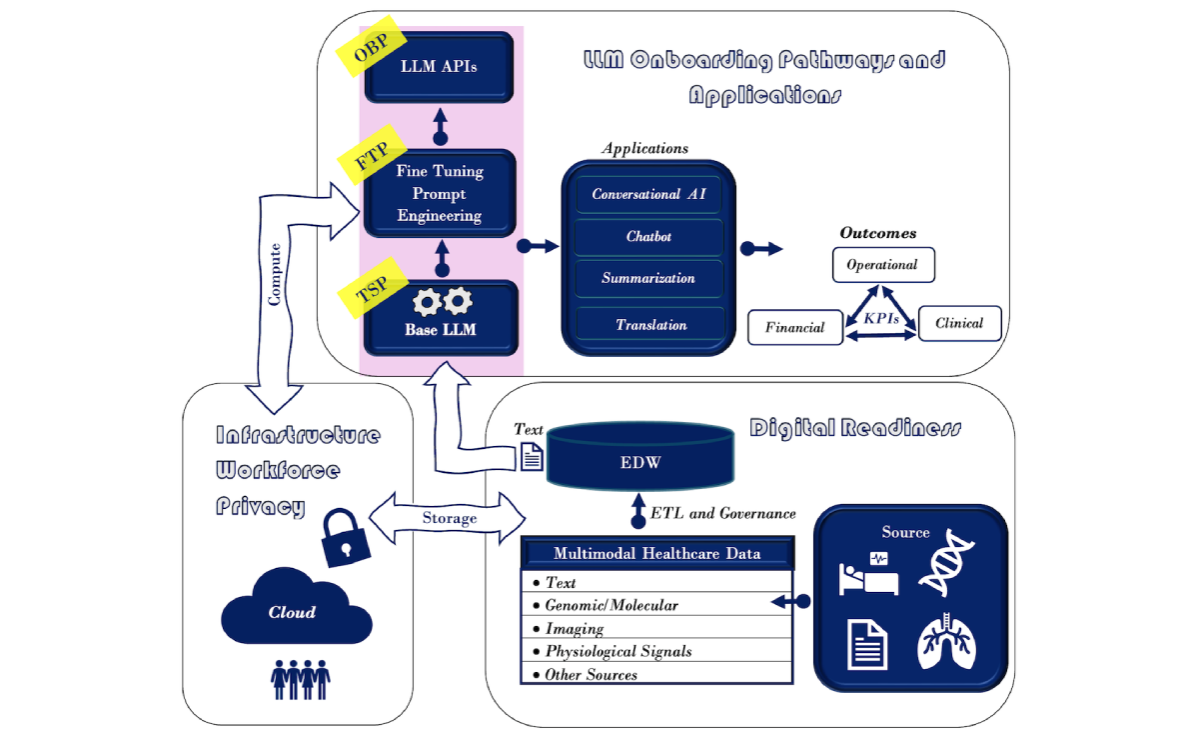
Essential ingredients for equitable distribution of large language models (LLMs) comprising 3 interconnected components: (1) digital readiness, (2) infrastructure workforce and privacy, and (3) LLM onboarding pathways (training from scratch pathway [TSP], fine-tuned pathway [FTP], and out-of-the-box pathway [OBP]) and applications to impact health care outcomes and key performance indicators (KPIs) in health care settings. AI: artificial intelligence; API: application programming interface; EDW: enterprise data warehouse; ETL: extract, transform, and load; MRI: magnetic resonance imaging.

The past decade has witnessed unprecedented growth and digitization of *multivariate* and *multimodal* health care data from diverse sources (eg, the electronic health record [EHR], claims, registries, Internet of Things, and molecular) [1,2]. While multivariate data represent data of a given type across a set of entities (eg, text), multimodal data represent distinct types of data (eg, text, image, and genomics) across entities of interest and play a critical role in generating comprehensive patient and population profiles. Multimodal health care data fall under 2 broad categories, namely *structured* (eg, diagnosis codes and the *International Classification of Disease*, ninth and tenth revisions) and *unstructured* data (eg, text and image). About 80% of health care data are unstructured, including text from clinical narratives [3,4]. The prevalence of unstructured textual clinical data is perhaps a primary motivating factor behind the continued evolution and adoption of natural language processing (NLP) [5] approaches for gaining novel insights from these datasets [6-8]. More recently, advanced machine learning techniques, such as deep learning (DL) [9-11], large language models (LLMs) [12,13], and foundation models, have accelerated these efforts with enhanced capabilities in deciphering patterns from unstructured data [14-16]. Multimodal health care data are usually extracted, transformed, and loaded (extract, transform, and load [ETL]) from diverse source systems into an enterprise data warehouse (EDW; Figure 1). Several variants (eg, Data Lake house) have also been proposed [17]. Subsequently, textual data from EDW are retrieved in a context-specific manner for downstream analytics and ingestion by LLMs (Figure 1). LLM implementations are governed by needs as well as affordability. This perspective discusses factors that impact LLM implementation and proposes 3 broad LLM onboarding pathways for its equitable distribution and adoption.

Multimodal digital footprints (Figure 1) capture unique characteristics of a given population with the potential to assist in decision-making in an evidence-based and data-driven manner, impacting outcomes and key performance indicators (KPIs). These outcomes typically fall under 3 broad categories (*clinical*, *operational,* and *financial*) that are not necessarily independent. For instance, data-driven approaches that can improve preventive care use can minimize aggressive disease-impacting clinical outcomes. Improved clinical outcomes can enable optimal resource allocation impacting operational outcome, reducing the economic burden on the patient, provider, as well as the payer impacting financial outcome. Therefore, the outcomes are represented by bidirectional arrows in Figure 1. While there is considerable excitement over the transformative potential of LLMs in health care [18], it is accompanied by significant *economic* challenges impacting their equitable distribution across health care organizations, especially those that serve economically disadvantaged communities. The choice of a particular LLM implementation pathway is dictated by the *needs* as well as *affordability*. In this perspective, 3 different LLM implementation pathways (training from scratch pathway [TSP], fine-tuned pathway [FTP], and out-of-the-box pathway [OBP]; Figure 1) across 4 major cloud service providers (CSPs; Amazon Web Services [AWS], Google Cloud Platform [GCP], Azure: Microsoft, and Oracle Cloud Infrastructure [OCI]) are presented as “onboarding points.” The risks, benefits, and economics of these pathways are presented and expected to assist in choosing a pathway and subsequent migration across pathways.

### Artificial Intelligence

**Figure 2:**
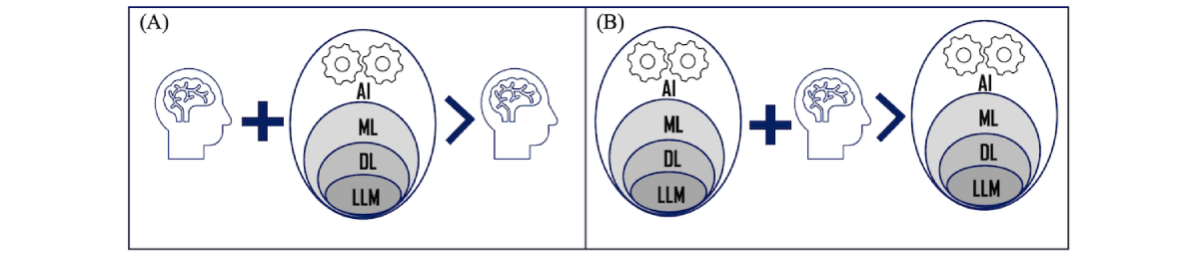
Augmented intelligence along with hierarchical representation of artificial intelligence (AI), machine learning (ML), deep learning (DL), and large language model (LLM) is shown in panels (A) and (B), respectively. The primary driver is shown to the right of the inequality in each of the panels.

Operational definition of artificial intelligence (AI) relies on the Turing test, which emphasizes the ability of computers to imitate humans in performing certain tasks [19]. These include automated reasoning, machine learning (ML), and NLP [19]. The hierarchical relationship of AI, ML, DL, and LLMs is shown in Figure 2. In contrast to classical statistical hypothesis testing, AI, ML, or DL assist in discovery, hypothesis generation, and validation in an evidence-based and data-driven manner, with LLMs exhibiting emergent abilities. ML is a branch of AI with a focus on the ability of machines to learn patterns from experience for a given task in an automated manner and draw inferences on previously unseen instances. Popular ML approaches with health care applications include supervised learning, unsupervised learning, reinforcement learning, and association mining [20,21]. DL [22-25] is a subfield of ML that specifically uses neural networks with multiple hidden layers to capture patterns of varying resolution in the given data. Unlike traditional ML, model parameters in deep neural networks (DNNs) can be considerably large with significant storage and computational demands. For example, DL models, such as transformers [26], an essential ingredient of LLMs, have billions of parameters [27]. The layered architecture of DNNs has also been shown to support “transfer learning,” where an existing neural network trained on a large dataset can be reused as a base network for predictions on related datasets by retraining only a subset of the layers. This is in stark contrast to traditional, shallow ML models where all model parameters may be altered upon retraining. Recently, proposed foundation models [5,14], exploit the transfer learning ability of DNN, where a “base” model trained on large multimodal data is subsequently adapted for various downstream tasks. LLMs use transfer learning in conjunction with augmented intelligence for enhanced performance, as discussed in the subsequent sections.

### Augmented Intelligence

Augmented intelligence emphasizes the importance of AI and humans working in concert for enhanced performance and generalization ability (Figure 2). The fundamental theorem of biomedical informatics by Friedman [28] emphasized the importance of information resources assisting domain experts by complementing the knowledge of the domain expert. In the present context, it essentially addresses the question, “Can domain experts in partnership with an AI resource lead to better insights than those unassisted?” (Figure 2A). As noted earlier (Figure 1), health care data are typically large, high-dimensional, multimodal, ingested from diverse sources and evolving rapidly, challenging manual interpretation. Therefore, AI models can assist in gaining novel insights from these datasets in an evidence-based manner while validating what is already known. The primary driver in Figure 2A is the domain expert with AI assisting the knowledge discovery process. On a related note, AI models implicitly subscribe to optimizing a chosen objective function and may converge prematurely to a local optimum. Several factors dictate the convergence aspects of these models [29]. This raises the question, “Can AI models with feedback from domain experts perform and generalize better than those unassisted?” (Figure 2B). More specifically, the role of the domain expert can assist in narrowing the set of variables in a context-specific manner, significantly reducing the search space of potential solutions and improving the performance and generalization ability while imposing necessary guardrails for optimal performance. Unlike Figure 2A, the primary driver in Figure 2B is AI, with the domain expert assisting in the knowledge discovery process. LLMs incorporate human feedback (reinforcement learning with human feedback) in minimizing bias, toxicity, hallucinations with improved performance, and generalization ability [30].

### LLMs in Health Care

#### Factors Impacting LLM Implementation

This section discusses three critical factors accompanying successful LLM implementations and deployments in health care settings: (1) *digital readiness,* (2) *infrastructure and workforce*, and (3) *privacy, ethics, and regulatory aspects* (Figure 1). These factors are closely related to the analytics maturity of a health care organization (eg, Healthcare Information and Management Systems Society Adoption Model for Analytics Maturity) [31], and their impact varies across the 3 LLM implementation pathways (TSP, FTP, and OBP).

#### Digital Readiness

Typically, LLMs (eg, LLaMA [Meta AI], GPT-4 [OpenAI], Med PALM-2, and Claude) are trained on massive amounts of data (eg, TB) integrated from multiple data sources, including those available publicly [32,33]. Large datasets used to train LLMs often lead to sizeable models with billions of parameters with a direct impact on their performance [27], while marking the transition from language models to LLMs with emergent abilities. So, access to large digital health care data (Figure 1) is critical in developing LLMs with superior performance. However, accessing sensitive health care data (eg, protected health information [PHI] and personally identifiable information) to train LLMs poses significant privacy and security challenges. Health care data, such as clinical narratives, are primarily governed by regulations, such as Health Insurance Portability and Accountability Act (HIPAA) [34] in the United States and the General Data Protection Regulation (GDPR) [35] in Europe. HIPAA establishes national standards for the protection of individually identifiable health information by covered entities and their business associates. Similarly, GDPR, although a European Union regulation, is designed to protect the privacy of European Union citizens and residents and applies to all organizations regardless of location. Both HIPAA and GDPR impose clear regulations on the release and sharing of health care data, with civil and criminal penalties for violations. Addressing privacy and security challenges associated with accessing sensitive health care data for training LLMs demands a robust data management strategy. Currently, there are 2 key strategies that stand out in this context: *data deidentification* and *confidential computing* [36,37]. Data deidentification involves removing personally identifiable information, both direct and indirect, from datasets to reduce the risk of patient reidentification. This allows the use of clinical data for model training purposes without compromising patient privacy. The deidentification process involves techniques such as removing names, addresses, social security numbers, and other direct identifiers, as well as managing quasi-identifiers such as dates of birth, gender, and medical diagnoses. However, these identifiers could potentially be used in combination with other information to reidentify individuals [38,39]. Therefore, deidentification singularly is not devoid of reidentification risks. The granularity of the data upon adequate deidentification can also significantly impact what could be effectively inferred. A recent study by de Kok et al [40] elucidated some of the challenges and best practices for sharing health care data compliant with GDPR. Subsequently, 4 approaches were proposed [40] for sharing open health care data (*consent pseudonymized*, *no consent pseudonymized*, *no consent anonymized*, and *no consent cloud*). However, the sensitive nature of health care data sharing has a direct impact on the sample size for training and fine-tuning LLMs. In general, health care datasets are relatively smaller by several orders of magnitude compared with datasets used to train popular general-purpose LLMs. Recently, Jiang et al [12] pretrained their LLM (NYUTron) primarily on health care data comprising 4,112,249,482, nearly 4 billion words, resulting in a 109-million-parameter model. In a related yet independent study, Yang et al [41] pretrained their LLM (GATORTron) primarily on health care data comprising 82 billion deidentified clinical words, resulting in 3 different LLM models of varying sizes (baseline: 345 million parameters, medium: 3.9 billion parameters, and large: 8.9 billion parameters). It might not be surprising to note that the size of these LLMs was markedly smaller than general-purpose LLMs (eg, LLaMA: 65 billion, GPT-3: 175 billion, and Google Pathways Language Model: 540 billion) [27] by several orders of magnitude. Empirical studies on the scaling behavior of LLMs reported improved performance and emergent behavior with increasing number of parameters (ie, size of the model) [42,43], size of the data, and compute time [44]. However, sample size challenges are likely to persist for health care datasets. Recent efforts in boosting the sample size of the training data from LLMs have explored supplementing health care data from EDW with biomedical text from sources, such as PubMed [41]. Approaches, such as federated learning (FL) [45-48] can also assist in overcoming some of the sample size-related challenges. However, FL could be susceptible to data leakage, breach, and fair and equitable data representation [49].

Multimodal health care data (eg, text, images, and signals) are usually integrated from diverse enterprise source systems (eg, electronic medical record, registries, the Internet of Things, next-generation sequencing, and Picture Archiving and Communication System) and reside in a centralized EDW (Figure 1). EDWs support querying, reporting, and enterprise analytics. Data governance and ETL are essential ingredients of EDW implementation, dictating the quality and granularity of the data ingested by downstream analytics tools, such as LLMs, impacting their performance. Data governance and ETL can also exhibit marked variations across health care organizations attributed to several factors, including variations in source systems and business processes. More importantly, EDWs demand upfront investment, continued support from executive leadership, and an existing, evidence-based culture. EDW economics is governed by several factors, including (1) architecture of EDW (eg, Inmon [50] and Kimball and Ross [51]); (2) source systems; (3) type, velocity, and volume of data; (4) enterprise data governance and ETL processes; (5) infrastructure and workforce supporting secured storage and retrieval; and (6) existing culture of data-driven and evidence-based approaches in impacting outcomes and KPIs in the health care organization. It is important to note that ETL and EDW can exhibit marked variations across organizations, posing challenges in seamless sharing of health care data, deployment of AI and ML models, and demonstrating their generalization ability. Common data models, such as the Observational Medical Outcomes Partnership, have been helpful in data standardization, data sharing, and federated querying [52,53]. However, there are inherent limitations to standardization, including incompleteness in data models and terminologies resulting in data that cannot be mapped [54], errors in mapping [55], and the potential loss of information due to granularity mismatches between the source data and the standard. In addition, data standards require significant expertise and a steep learning curve in their absence. The challenges mentioned earlier can especially be accentuated across health care organizations that primarily serve the economically disadvantaged, underserved, and marginalized communities. Factors contributing to data inequities [56] are multidimensional and include ethnicity (eg, Hispanic), race (eg, African American), disease groups and treatment regimens (eg, rare disease), gender or gender identity (eg, lesbian, gay, bisexual, transgender, and queer [LGBTQ]), age (eg, pediatric population), geographic location (eg, rural areas), language barriers (eg, Spanish), digital divide, and patient literacy [57,58]. It is of interest to note that these dimensions are not mutually exclusive, and their combination can significantly impact the representation of a given population in the data and digital readiness of organizations that serve these communities. Inequity along these dimensions can lead to potential biases [59,60]. These biases broadly fall under *systemic*, *statistical*, *computational,* and *cognitive* bias [61]. Systemic bias can be further categorized into *measurement bias*, *missing validation bias*, *label bias,* and *modeling bias* [61]. Measurement bias [62,63] may be the result of variations in quality and representation of the entities of interest across subpopulations in the data. Missing validation bias may result due to a lack of adequate validation studies across certain populations. It is important to note that measurement bias may accentuate validation bias because validation at small sample sizes can be statistically challenging. Label bias may be a result of surrogate variables substituting the actual health care outcomes of interest. Modeling bias is attributed to the biased results generated by a specific model. Computational and statistical biases can be a result of inadequate representation of select groups and populations in the given data. Human cognitive bias is a bias due to human perception of AI and ML systems. Mitigating human cognitive bias may be critical for successful adoption of AI systems. From this discussion, digital readiness is critical for health care organizations to embark on their LLM journey and could dictate the choice of the LLM implementation pathway.

#### Infrastructure and Workforce Needs

##### Infrastructure Needs

LLMs primarily rely on the transformer architecture [26], with the ability to be trained in a massively parallel manner on sequential data. However, parallel processing in turn demands specialized hardware accelerators, such as graphics processing units (GPUs), and seamless interfaces between various hardware components in conjunction with significant network bandwidth for avoiding storage, data transfer, latencies, and bottlenecks (Figure 1). This is especially true when the training involves large amounts of data and pretrained LLMs have billions of parameters. Parallelism falls under 2 broad categories, namely (1) *data parallelism* [64] and (2) *model parallelism* [65]. Data parallelism addresses challenges with large training datasets that cannot fit within a single GPU by partitioning and distributing them across multiple GPUs. This is especially helpful because optimization techniques, such as stochastic gradient descent, used by LLMs rely on small batches of data that can be distributed across GPUs. Model parallelism addresses challenges with the size of the LLMs (eg, billions of parameters) by distributing them (eg, weights and layers) across multiple GPUs and further subdivided into *pipeline parallelism* and *tensor parallelism*. While pipeline parallelism facilitates the distribution of the layers of a DNN [66], tensor parallelism [67] distributes the tensor computation across hardware accelerators. While partitioning the data and model is useful in overcoming some of the challenges with their size, fast interconnect between the GPUs is especially critical for enhanced communication between them in a cluster (eg, DGX A100 Data Center) and between clusters (eg, Super PODs) through specialized high-bandwidth (eg, 900 GB/s) communication links, such as NVLink/NVLink Switch. Training LLM models using multiple GPUs is also accompanied by significant carbon footprint and heating, demanding specialized cooling systems for optimal performance [68]. While LLM implementations are traditionally dependent on multiple software libraries [69], there has been recent interest in developing graphical user interfaces for LLMs to alleviate some of these challenges for the end users [70]. The size of the GPUs required is dependent on several factors, such as the size of the LLM models so that a model can be loaded into the GPU successfully while permitting necessary computation. For instance, LLM models with 7 billion parameters (13 GiB) may need a 16-GiB GPU, such as NVIDIA T4 Tensor Core for processing, while those with 13 billion (25 GiB) and 70 billion parameters (130 GiB) might require a 32 GiB (eg, NVIDIA-A100 GPU Server) and 160 GiB GPUs (eg, NVIDIA 2 × A100 multi-GPU Server), respectively.

##### Cloud-Computing Platforms

On-premise infrastructure had supported more traditional AI and ML implementations and analytics dashboards in the past. However, increasing size of the data and compute along with evolving needs of health care organizations, demand scalable infrastructure for storage (horizontal scaling) and computing (vertical scaling), with the latter playing a critical role for LLMs. For instance, data and model parallelism demand scalable infrastructure that can vary with training data size as well as the model size. Cloud-computing environments (Figure 1) provide infrastructure as a service and software as a service (SaaS) with pay-as-you-go payment models to address challenges from scalability, privacy, workforce, and economic standpoints. CSPs (AWS, GCP, Azure, and OCI) offer robust solutions for LLM deployment by leveraging their global infrastructures built around *high availability*, *enterprise security and compliance*, *low latency* access to computing resources, and managed services as needed. CSPs can play a critical role in bridging the chasm between needs, such as growth scale and security of information technology infrastructure, and affordability by leveraging existing technology investments on-premises or on other clouds. More importantly, CSPs can help facilitate enhanced democratization and equitable adoption of LLMs by the broader health care communities and not by a privileged few, a critical aspect for the equitable distribution, widespread adoption, and long-term success of LLMs in health care. CSPs also provide hybrid options, such as “cloud bursting,” that allow organizations to use their private cloud, on-premise infrastructure, for routine operations and burst into a CSP temporarily when additional computing resources are needed to handle peak loads and prevent queuing of computational workloads. A hybrid approach enables health care organizations to handle the variable and intensive computational demands of LLMs at scale. By cloud bursting, health care organizations can maintain a cost-effective private cloud for steady-state workloads and then burst into public clouds during periods of high demand without needing to overprovision their private cloud infrastructure while minimizing latencies. CSPs provide essential tools to help organizations manage security and compliance risks. AWS identity access management and Azure controls are examples of services that integrate into existing organizational security investments and aid in configuring fine-grained access controls and ensuring authorized access to sensitive data. Hybrid cloud offerings from AWS, GCP, and Azure also adhere to regulations and compliance (eg, HIPAA) for handling health care data containing PHI through data encryption and network security between on-premises and on-cloud workloads, creating a single flexible, cost-effective enterprise infrastructure technology solution. These environments can also assist in minimizing on-premises workforce needs and advanced skillset needs using readily available cloud-based tools and managed services. This aspect is especially critical for health care organizations that do not have sufficient up-front investment and a history of supporting analytics teams but would like to experiment with the utility of LLMs for impacting outcomes. In essence, CSPs can assist in equitable adoption by starting small with existing resources and scaling out as needed without large upfront investments. Utility services from CSPs can significantly reduce the operational overhead and technical challenges associated with LLM development, testing, and scale, allowing for health care organizations to focus more on outcome-based applications rather than infrastructure management [71]. Serverless options by CSPs for storage and computing are also expected to minimize carbon footprints [68].

##### Cloud-Based Hardware Accelerators

Hardware accelerators assist in overcoming data and computational bottlenecks working in concert with base processors (eg, central processing units) [72]. Several types of accelerators, such as application specific integrated circuits, field programmable gate arrays, GPUs, and dedicated chips for AI, have been explored [73-76]. Accelerators, such as GPUs, as noted earlier, have become an integral part of DL models and LLMs in overcoming computational bottlenecks. However, there is renewed interest in developing hardware accelerators for LLM training and inference that are affordable. Hardware accelerators offered by CSPs include Trainium and Inferentia (AWS), tensor processing units (TPUs; GCP), Maia (Azure), and MI300X (OCI). These chips are optimized for specific workloads, cost-effective relative to GPUs, and interface to popular open-source environments. However, multiple factors, such as the choice of open-source environment and machine-learning libraries (eg, Tensorflow and PyTorch), can impact the benchmarking and performance of hardware accelerators [77].

- AWS—Trainium AI accelerator [78] supports training DL models with >100 billion parameters and up to 50% in cost-to-train savings over comparable elastic compute cloud instances. Inferentia [79] supports inference, delivering up to 2.3× higher throughput and up to 70% lower cost per inference than comparable elastic compute cloud instances. Both are supported by the AWS Neuro software development kit, which integrates natively with open-source DL environments such as PyTorch, TensorFlow, and HuggingFace. Inference on Meta’s llama 8B would cost US $0.99/h on Inferentia, a savings of >65% on an NVIDIA A10G GPU instance.
- GCP—TPUs [80] are custom-designed AI accelerators optimized for training and inference of AI models [81]. They scale cost-efficiently for a wide range of AI workloads, spanning training, fine-tuning, and inference. TPUs provide the versatility to accelerate workloads on leading AI frameworks, including PyTorch, JAX, and TensorFlow.
- Azure—Maia 100 AI accelerator [82] is supported by the Maia software development kit and interfaces to open-source frameworks such as PyTorch, ONNX Runtime, and Triton from OpenAI. It can support services such as Microsoft Copilot and Azure OpenAI Service.
- OCI—MI300X accelerators are powered by AMD’s CDNA 3 architecture, offering memory bandwidth and compute performance supporting a broad range of precision data that enable OCI to support larger and more complex computations for AI and ML workloads [83-85]. Compared with NVidia’s H100GPUs, MI300X could provide a cost-effective alternative while still delivering competitive performance with its 304 compute units, 19,456 stream cores, and 1216 Matrix cores.

##### Cloud-Based Managed Services

Managed services from CSPs can assist in LLM implementation and management while minimizing on-premises workforce needs. Managed services features can be readily accessed through application programming interfaces (APIs), accelerating implementations with an enhanced focus on impacting health care outcomes. This may include access to multiple LLMs across vendors via a single API, enabling experimentation by end users without managing multiple end points, keys, and payloads. This permits experimentation with new LLM models and automates this process via CSP-managed services. These features are especially helpful in exploring the available LLMs that best suit the current needs of the health care organization. In contrast, the exploration phase could be fairly involved across on-premise implementations, accompanied by multiple tokens, API end points, and access controls. CSP-managed LLM services also facilitate centralized governance structures for access management, billing, auditing, and security scanning. Given the sensitive nature of health care data, using fewer end points allows health care organizations to set up the necessary access controls for data and APIs in a seamless manner.

##### Workforce Needs

LLM implementation and deployment in health care workflows typically demands a workforce with expertise across a spectrum of areas (Figure 1). However, the workforce needs will be dependent on several factors that include (1) LLM implementation pathway, (2) applications of LLM in health care workflows, (3) commercial or open-source platforms. and (4) on-premise or cloud-based implementations. Workforce needs are especially critical across LLM implementations that involve training and fine-tuning. This would ideally consist of a (1) core team comprising data scientists, architects, engineers, and CSPs with expertise in areas such as warehousing, NLP, LLMs, ML implementation, deployment, and assessment (eg, MLOps) [86] in concert with (2) an infrastructure team that addresses storage and computing needs on-premises and on-cloud, (3) subject matter experts and health care personnel who guide the implementation, reinforcement learning, and prompt engineering aspects of LLMs for optimal performance, (4) regulatory and compliance teams for ensuring ethical use of health care data and establishing guardrails, and (5) information technology that assists in ensuring privacy and security of health care data while deploying the LLM applications or API in enterprise workflows to impact outcomes and KPIs (Figure 1). Agile strategies (eg, DevOps or MLOps) may be critical for implementation, validation, and seamless deployment of LLMs in workflows. Given potential bias and toxicity that may accompany LLM implementations, an inclusive framework that incorporates critical feedback from stakeholders, patients, providers, and subject matter experts across diverse communities can minimize bias and assist in developing the necessary guardrails.

#### Privacy, Ethics, and Regulatory Aspects

##### Privacy and Security in the Cloud

Privacy and security are critical for storage, retrieval, and analysis of health care data (Figure 1). There has been an increasing shift in moving health care data and analytics from on-premises to the cloud [71]. CSPs provide confidential computing environments (CCEs) that facilitate computations in hardware-based trusted execution environments (TEEs). These ensure sensitive data (eg, PHI data) to remain encrypted in an isolated environment, preventing modification of data and applications by unauthorized parties, including CSPs, during processing and transmission [87,88]. They facilitate secure collaboration of first- and third-party data with the potential to assist in overcoming sample size constraints for LLM training as discussed in this section. CCE capabilities across the 4 major CSPs (AWS, GCP, Azure, and OCI) are discussed in the subsequent sections.

- AWS—Confidential computing capabilities through the processor agnostic AWS Nitro System and AWS Nitro Enclaves, enabling secure isolation of sensitive workloads [89].
- GCP—Confidential virtual machines (VMs) and Confidential Google Kubernetes Nodes allow customers to process sensitive data while keeping them encrypted in memory [90].
- Azure—Confidential computing VMs with AMD SEV-SNP and Intel SGX support ensuring VM-level confidentiality and protection from cloud operators [91].
- OCI—Confidential computing through Oracle’s confidential instances leveraging AMD Secure Encrypted Virtualization for VMs and AMD secure memory encryption for bare metal instances protecting data and application processing the data [92].

##### Attestation

Attestation is a critical component of CCEs that ensures the trustworthiness of the computing environment. It allows the integrity and authenticity of the hardware, software, and configuration to be verified, effectively establishing trust between parties. Attestation offered by major CSPs (AWS, GCP, Azure, and OCI) is discussed in the subsequent sections.

- AWS—CCE attestation involves the use of an attestation document signed by the Nitro Hypervisor. This document is critical for providing the identity of the enclave to AWS Key Management Service (KMS), which validates the document against the KMS key policy. This allows the enclave to perform cryptographic operations with KMS keys [93].
- GCP—Provides attestation through the Binary Authorization and Certificate Authority Service. Through this service, the confidential workload collects measurements of itself and the TEE and sends an attestation request to the Binary Authorization service, which compares the measurements against an attestation policy. If they match, service returns a signed attestation [94].
- Azure—Provides CCE attestation via the Microsoft Azure Attestation service. The confidential workload includes an attestation client that collects measurements and evidence from the TEE. It then sends an attestation request with its evidence to the Microsoft Azure Attestation service that is verified against policy. If valid, it returns a signed attestation token.
- OCI—Provides attestation using a hardware-based trusted security module that generates an attestation report containing measurements of the hardware and firmware environment and verified by the customer to ensure confidential workload is running in a legitimate TEE.

### FL Architecture

CSPs also provide FL architectures [47] for decentralized training of LLM addressing sample size challenges. An FL model is trained locally and refined through shared updates, resulting in an aggregated global model without explicit sharing of health care data. Using federated data for training may leverage collective knowledge, perhaps resulting in models with enhanced generalization ability. It is important to note that because only model updates are shared instead of the actual data, FL implicitly minimizes the amount of data transferred over the network. This can be particularly beneficial in scenarios where data transfer is costly or limited by bandwidth constraints. FL frameworks developed by CSPs include:

- AWS—FedML on AWS is an open-source library that supports several FL models. AWS provides tools, libraries, and algorithms to implement and experiment with FL algorithms in a private and secured environment [95].
- GCP—TensorFlow Federated is an open-source framework for ML on decentralized data. TensorFlow Federated is used to implement FL on Google Cloud, leveraging Google Kubernetes Engine for hosting and managing the FL process [96].
- Azure—the AzureML platform supports Azure FL frameworks NVFlare and Flower for running a FL pipeline. Azure’s capabilities are leveraged for provisioning and orchestration of FL algorithms [97].
- OCI—Supports FL through various tools, frameworks, and services, such as Private Federated Learning with Domain Adaptation [98].

### Confidential FL

While FL ensures compliance with data protection regulations, such as HIPAA and GDPR, it does have some limitations, as noted earlier [49]. A possible solution is to combine confidential computing and FL, resulting in confidential FL (CFL) [99]. This decentralized approach works well for a hybrid cloud environment that spans on-premise data centers, edge devices, and public clouds from different CSPs. Confidential computing TEEs secure the data during processing, while FL enables collaborative training without explicit sharing of health care data. Incorporating a deidentification process as a part of CFL workflow ensures access to large sensitive data for training LLMs without compromising privacy and security while maintaining the integrity of health care data management. Key characteristics of CFL across CSPs are discussed in the following sections.

- Enhanced privacy and security—while deidentification removes identifiable information from the data, CFL ensures that the data are processed in a secure and private manner.
- Compliance or regulations—Deidentification and CFL can help health care organizations comply with HIPAA and GDPR. CFL provides the necessary security measures to protect data in use. Both technologies address regulations regarding data security.
- Facilitation of data sharing—by combining these technologies, health care organizations can safely engage in collaborative data sharing initiatives and develop LLM models with enhanced performance and generalization ability.
- Intellectual property protection—CFL can protect intellectual property such as health care AI algorithms and research data during collaborative training.
- Building trust—secure handling of health care data builds trust among patients, providers, and payers.

While CFL has the potential to accelerate LLM implementation using sensitive federated health care data, some of the challenges listed here need to be addressed for its successful deployment and adoption.

- Data heterogeneity—CFL assumes that the data across participating organizations are independently and identically distributed. However, in practice, health care data may exhibit significant heterogeneity, which can impact the performance of the models. Techniques, such as transfer learning and domain adaptation, can be used to address this challenge.
- Communication efficiency—the iterative nature of CFL involves frequent communication between the participating organizations and the central data server. Efficient communication protocols and compression techniques are necessary to minimize the communication overhead and ensure scalability.
- Model interpretability—CFL models may lack interpretability due to the distributed nature of the training process. Techniques, such as model distillation and explainable AI can be used to improve the interpretability of CFL models.
- Incentive mechanisms—encouraging health care organizations to participate in CFL initiatives may require appropriate incentive mechanisms. Developing fair and transparent incentive approaches that align with the interests of all stakeholders is an important consideration for success.
- Human in the loop—integrating human expertise and oversight as a part of the LLM training and decision-making process ensures that the models are accurate, reliable, and aligned with human values. Human in the loop also ensures that the models comply with legal and regulatory requirements, such as data protection laws and medical standards.

### Ethics and Regulatory Aspects

The potential of AI tools, such as LLM, to transform health care outcomes does come with various ethical and regulatory challenges [100-102]. US president Joe Biden’s October 2023 executive order [103] underscored the necessity of ensuring AI safety and security. It mandated AI-generated content to be clearly identified and called for substantial investments in AI-related education, training, and research. The order emphasized protecting intellectual property, supporting American workers, advancing equity, civil rights, while safeguarding privacy and civil liberties. It also directed the Department of Health and Human Services to establish safety parameters for AI, including frameworks for identifying and tracking clinical errors, generating improvement guidelines, and sharing these among health care organizations. A recent Health and Human Services 2024 ruling (Section 1557, Patient Protection and Affordable Care Act) also emphasized protection to patients against bias and discrimination from AI and ML decision support tools and the importance of mitigating such biases [104,105]. In addition, 29 countries attended the AI Safety Summit in November 2023 and signed the Bletchley Declaration [106] to “cooperate on AI to promote inclusive economic growth, sustainable development, and innovation, to protect human rights and fundamental freedoms, and to foster public trust and confidence in AI systems to completely realize their potential.” The Institute for Healthcare Improvement’s Lucian Leape Institute (LLI) [107] predicted increased use of AI in clinical documentation support, clinical decision support, and patient-supportive chatbots in the health care setting. They recommended prioritizing patient safety, engaging clinicians, ensuring AI efficacy and bias mitigation, establishing AI governance and oversight, and fostering collaborative learning across health systems. In addition, LLI emphasized the importance of AI systems in reporting confidence levels and rationale and the need for continuous human monitoring to maintain trust and accuracy in AI-generated outputs. LLI also suggested several considerations for regulators and policy makers: establishing clear guidelines for ethical and trustworthy AI use, promoting transparency and accountability, supporting AI literacy, incentivizing AI development that prioritizes safety, and facilitating localized decision-making. The European AI Act [108], the first legal framework on AI, categorized AI risks into 4 levels—unacceptable, high, limited, and minimal—and introduced transparency obligations for all AI models. Accreditation agencies, such as the Joint Commission, will need to advocate to create governance structures and processes for monitoring patient safety issues related to AI. The World Health Organization also recently commented on regulatory considerations for AI in health [109]. It outlined essential guidelines covering documentation and transparency, risk management, intended use validation, data quality, privacy protection, and stakeholder engagement. The ethical development of AI must adhere to principles, such as beneficence, nonmaleficence, autonomy, justice, data quality, transparency, fairness, responsibility, privacy, freedom, trust, sustainability, dignity, and solidarity. Trustworthiness of AI-based clinical decision support is often compromised by the lack of transparency in how AI tool’s function and the basis of their decisions. There are concerns about the use of proprietary data, the absence of robust regulation, and the risk of bias from datasets that do not adequately represent marginalized populations. Overall, as AI continues to evolve and integrate into health care, maintaining a balance between innovation and ethical responsibility is crucial. Regulatory frameworks and ethical guidelines at health care organizations must evolve to ensure that AI enhances health care delivery while protecting the interests and rights of patients and providers alike.

### LLM Guardrails for Responsible AI

There has been interest in developing guardrails and regulatory frameworks to facilitate responsible AI. These guardrails ensure the behavior of AI tools, such as LLM, falls within expected bounds while being resilient to adversarial attacks. These efforts include recent open-source initiatives, such as NeMo Guardrails [110] for improved trustworthiness [111] of LLM conversational systems. These guardrails assist in customizing the LLM interaction with users using *topical rails* and *execution rails* [110]. While topical rails prevent the LLM from veering off topic, execution rails assist in moderating the LLM output and ensure it is factual. A recent study by Meskó and Topol [102] on regulatory oversight of LLMs identified several associated challenges. The study pointed out the challenges in translating existing Food and Drug Administration’s regulatory frameworks for medical devices [112] to contemporary AI-based technologies, such as LLMs, and the need for new regulatory frameworks for LLMs. More specifically, it highlighted 2 unique characteristics of LLMs: (1) the ability to adapt their performance to training data as well as tasks in contrast to traditional AI and ML approaches, and (2) the ability to learn in a self-supervised (autodidactic) manner without the need for explicit guidance and ground truth labels as in a more classical supervised ML paradigm. Subsequently, a list of LLM regulatory challenges were identified (Table 1 in the study by Meskó and Topol [102]), including privacy, intellectual property, medical malpractice liability, quality control and standardization, informed consent, interpretability and transparency, fairness and bias, data ownership, overreliance, and need for continuous monitoring and validation. Some of these challenges have also been acknowledged in a more recent US Food and Drug Administration release with a broader focus on AI and medical products [113].

**Table 1:** Summary of risks and benefits of the 3 large language models (LLMs) onboarding pathways (training from scratch pathway [TSP], fine-tuned pathway [FTP], and out-of-the-box pathway [OBP]).

		TSP	FTP	OBP
**Digital readiness**				
	Benefits	LLMs are trained on health care data and capture characteristics of that target population with the potential to impact outcomes in that population. Enhanced transparency of the data, implementation, and deployment. Enhanced quality of training data through enterprise governance, minimizing bias.	Digital readiness of FTP is much lesser than TSP because FTP focuses on fine-tuning as opposed to training.	Digital readiness for OBP is minimum.
	Risks	Demands upfront investment in data warehousing, enterprise governance, and dedicated workforce.	Susceptible to biases in training data used in the pretrained LLMs. General purpose LLMs are often trained on nonhealth care data. Prompt engineering demands can be significant.	General purpose, out-of-the-box models pretrained on nonhealth care data may have limited utility, prone to bias, and hallucinations.
**LLM**				
	Benefits	Train LLM from scratch using either existing transformer architectures or novel architectures. Long-term maintenance, customization, with evolving needs.	Uses off-the-shelf pretrained LLMs without explicitly training from scratch. The number of open-source pretrained LLMs continues to grow.	Multiple choices of off-the-shelf LLMs accessed as APIs^a^. Readily accessible with minimal training.
	Risks	Cost of training LLMs can be significant. Sharing checkpointed LLMs trained on PHI^b^ data is a risk. Novel architectures demand considerable experimentation for optimal performance. May result in implementation and deployment delays.	Susceptible to biases in the pretrained LLMs. Pretrained LLMs on nonhealth care data may have performance limitations. Dependency on pretrained LLMs is a risk. Limited transparency may be a security risk.	Use is dependent on the features exposed by the vendors. Generic nature of the output may have limited utility in addressing the unique needs of health systems. No explicit training of the LLM model.
**Workforce**				
	Benefits	On-premise workforce can assist in customizing LLMs with enhanced transparency and evolving needs.	Workforce demand is significantly less than TSP.	Minimal on-premise workforce needs and training. Rapid implementation using managed services.
	Risks	Demands a skilled workforce with expertise across a spectrum of areas for implementation and deployment. Demands recruitment, growth, and retention of skilled workforce. Continued buy-in from leadership for sustaining workforce.	Limited customization of the pretrained LLMs.	Complete dependence on vendor models and services with minimal transparency
**Infrastructure and security**				
	Benefits	Options for training LLMs across cloud service providers with on-demand and spot instance pay-as-you-go pricing models. Secured cloud environments are available	Infrastructure needs for compute are significantly less than TSP.	Infrastructure is needed primarily for inference.
	Risks	Training LLMs on GPUs^c^ is expensive. Security and governance for training sensitive data in the cloud.	Vendor pricing may vary based on adoption. Prompt engineering on pretrained models can be involved.	Infrastructure costs increase with the number of users. Vendor pricing may vary with increasing adoption. Availability of services is not guaranteed. Interfacing to downstream applications.

^a^API: application programming interface.

^b^PHI: protected health information.

^c^GPU: graphics processing unit.

### LLM Implementation Triumvirate

#### Overview

As noted earlier, LLM implementation is dependent on several factors. In this section, 3 broad LLM implementation pathways (Triumvirate) are discussed along with associated risks, benefits, and economics (Figure 1). These pathways are not necessarily independent and expected to serve as onboarding points for equitable distribution and enhanced adoption of LLMs. A summary of the risk and benefits of these 3 pathways is also enclosed in Table 1 for convenience.

#### The TSP

In TSP, an LLM is trained from scratch using health care data and subsequently customized for specific needs and tasks of the health care organization. FTP and OBP may follow TSP.

#### Benefits

The TSP provides enhanced transparency of the data and code, complete ownership of the model parameters, implementation, and the ability to assess the quality of the training data at the most granular level. TSP is expected to facilitate long-term maintenance of the model and customization to the evolving needs of the health care organization, including seamless deployment in workflows and interface to enterprise dashboards. In addition to using an established LLM architecture, TSP may also implement novel architectures or modifications to existing general-purpose transformer architectures. Training LLMs using data from the EDW of a health care organization implicitly adheres to the data governance and ETL ensuring high-quality data and accommodating characteristics of the population served by the health care organization that may not necessarily be captured in generic datasets, such as those used to train general purpose LLMs. This in turn is expected to result in a model with enhanced performance [114] addressing the needs of the organization impacting clinical, operational, and financial outcomes and KPIs (Figure 1). Such a model is also expected to be better used while demonstrating value because the training data can significantly impact its behavior and performance [115]. As noted earlier, it is not uncommon for the organizational data to be supplemented by high-quality external data during training [41]. Unlike pretrained LLMs, access to the training data and enhanced transparency may assist in mitigating biases, minimizing perpetuation and amplification of biases, and reducing toxicity by the model as well as downstream applications and APIs that are dependent on the base model (Figure 1), leading to improved overall performance. This aspect is especially critical when deploying the LLM model in clinical workflows to assist in clinical decision-making.

#### Risks

TSP implicitly demands digital readiness, infrastructure and workforce, and regulatory compliance. Because sample-size challenges can impact TSP, approaches and FL techniques may be explored. The digital and analytics maturity of an organization, along with an existing culture of data-driven and evidence-based approaches to impacting outcomes, may be critical for successful TSP implementation and continued buy-in from the enterprise leadership. Unlike pretrained LLMs, TSP may demand experimentation to identify the optimal model size, parameters, and checkpointing the model before deployment. Agile implementation strategies across multiple teams, such as data science, information technology, clinical, and support from executive leadership may be critical for the timely progress of TSP. Therefore, timelines for TSP implementation and deployment are expected to be significantly larger than FTP and OBP. In addition to implementation and validation, timelines would also include seamless deployment in health care workflows and providing necessary training for the end users. Delays in demonstrating value are to be expected as with any new AI tool. TSP will also demand access to specialized infrastructure for storage and computing, including distributed frameworks and GPU Clusters or PODS. Enterprise CSPs can be critical partners in this regard. Unlike general purpose LLMs, TSPs using cloud infrastructure should follow strict compliance and security protocols that in turn may incur additional costs. Because TSP demands unique skill sets for implementation and critical evaluation, existing skillsets and investment in the workforce in areas such as data science, DL, NLP, LLM, and IT, as well as protected time for health care personnel for critical assessment of the models, would be important. The quality and performance of TSP will be dependent on the knowledge of the subject matter experts assisting with the reinforcement learning with human feedback process. FTP is likely to follow TSP as a part of customizing the LLMs to end users and downstream applications (Figure 1). Given the large number of parameters of LLM models, there is the possibility of the LLM models memorizing some of the information in the training data [116]. This in turn may discourage sharing checkpointed TSP models due to the risk of information leak.

#### Economics of TSP

Among the 3 pathways, TSP demands considerable up-front investment with regards to digital readiness, infrastructure, workforce, privacy, and regulatory aspects. TSP implementation will demand (1) an existing EDW for querying and retrieval of unstructured data for ingestion by LLMs; (2) a workforce with competencies across a spectrum of areas, including implementation, integration as well as technical aspects in multiple areas, including DL; (3) concerted working of multiple teams, including subject matter experts for validation and prompt engineering; and (4) regulatory oversight because the training phase in TSP would involve using health care data with the DL algorithm. Data-warehouse implementation and continued management could cost millions of dollars. The cost of hiring and retaining a workforce to support LLM implementation can be substantial, especially given the high demand for such specialized skillsets. For a midsized health care organization, the cost of a workforce capable of handling LLM development, implementation, deployment, and maintenance can range from US $2 million to US $5 million per year, including salaries, benefits, and training. Training and deploying LLMs require significant computational resources, including high-performance storage and computing infrastructure. For example, DGX A100 data centers (80 GB) were priced at approximately US $200,000 in 2020. LLaMA implementation [32] required 2048 A100 GPUs and 21 days for training their 65 billion parameter model, resulting in significant costs in millions of dollars. Therefore, the cost of compute, along with power consumption, physical space requirements, and dedicated personnel, could easily reach into the tens of millions of dollars for TSP. Working in partnership with cloud platforms can address several of these challenges. Major CSPs, such as AWS, Azure, GCP, and OCI, offer on-demand compute instances across GPU clusters and cost profiles based on the user needs and affordability within a secured framework (Table 2) [117-122]. TSP is usually followed by FTP to tune the response of the LLMs.

Because TSP demands building an LLM using the health care organizations data, it may require *hundreds of thousands of hours* of training [123]. Comparable pricing of 320 and 640 GiB of GPU memory using 8 × A100 GPUs in a single instance across the 4 major CSPs is presented in Table 2. On the basis of the comparison table and the time for training an LLM from scratch, it might be economical to purchase a long-term (3-year commitment), which may save around 60% when compared with on-demand costs. Another option would be to use a “spot instance” (Table 3) [124-127]. Spot instances are spare compute capacity offered by CSPs at a reduced cost compared with the on-demand pricing and serve as a suitable alternative. These pricing estimates vary with demand and can change throughout the day, week, or month. However, to use spot instances for LLM training, organizations need to implement strategies to handle instance reclamations and checkpoint management. This is especially critical for TSP, as it takes considerable time to train an LLM from scratch. CSP-managed services offer managed spot training or fine-tuning and resume jobs from the checkpoints. Due to spot interruptions, training or fine-tuning using spot instances may also take longer to complete compared with on-demand or reserved instances. With increasing adoption of LLMs by health care organizations in conjunction with the popularity of AI and ML availing spot instances in general can be challenging and could be prone to interruptions with marked variations in availability as well as pricing across the different geographic regions. Spot instances can also vary across CSPs, with some (eg, AWS, GCP, and OCI) providing more options and higher-memory GPU instances.

**Table 2:** Comparable per-hour pricing of graphics processing unit (GPU) clusters across cloud service providers (CSPs; Amazon Web Services [AWS], Azure, Google Cloud Platform [GCP], and Oracle Cloud Infrastructure [OCI]) for 320 or 640 GiB GPU memory for the training from scratch pathway (TSP). Representative data retrieved on June 2024.

CSP	Instance type	CPU^a^ (cores)	Memory (GiB)	GPUs	Per GPU memory	Total GPU memory	On demand (US $)	On demand per GPU (US $)	1 year (US $)	3 years (US $)
AWS	p4d.24xlarge	96	1152	8	40	320	32.77	4.10	19.22	11.57
AWS	p4de.24xlarge	96	1152	8	80	640	40.96	5.12	24.01	14.46
Azure	ND96asr A100 v4	96	900	8	40	320	27.20	3.40	22.62	13.63
Azure	ND96amsr A100 v4	96	1900	8	80	640	32.77	4.10	20.97	14.42
GCP	a2-highgpu-8g	96	680	8	40	320	29.39	3.67	18.52	10.29
GCP	a2-ultragpu-8g	96	1360	8	80	640	40.22	5.03	—^b^	—
OCI	BM.GPU4.8	64	2048	8	40	320	24.40	3.05	—	—
OCI	BM.GPU.A100-v2.8	128	2048	8	80	640	32.00	4	—	—

^a^CPU: central processing unit.

^b^Not applicable.

**Table 3:** Comparison of per-hour spot-instance and on-demand pricing across cloud service providers (CSPs; Amazon Web Services [AWS], Azure, Google Cloud Platform [GCP], and Oracle Cloud Infrastructure [OCI]) large language models (eg, 320 GiB, 8 × A100 graphics processing units [GPUs] single instance) and smaller and medium large language models (eg, 64, 16 GiB V100 GPU). Representative data were retrieved in June 2024.

CSP	Large models				Medium and smaller models		
	Instance type	On-demand cost per hour (US $)	Spot cost per hour (US $)	Instance type		On-demand cost per hour (US $)	Spot cost per hour (US $)
AWS	p4d.24xlarge	32.77	8.37	p3.8xlarge		12.24	3.97
Azure	ND96asr A100 v4	27.20	8.19	NC24rs_v3		13.46	0.91
GCP	a2-highgpu-8g	29.39	11.75	n1-highmem-32		21.73	3.63
OCI	BM.GPU4.8	24.40	12.20	BM.GPU3.4		12.03	6.02

### FTP Overview

While TSP focuses on pretraining LLMs, FTP focuses on adapting an existing pretrained LLM with a given architecture and parameters to tasks at hand in a domain-specific manner. This is usually accomplished by (1) adjusting the model parameters of the LLM using context-specific data that are much smaller than the training data and (2) adjusting the LLM performance and behavior by prompt engineering inputs and outputs of the LLM. The pretrained LLMs can be either open-source or proprietary LLMs, with those pretrained on health care data expected to perform better than general-purpose LLMs.

#### Benefits

The timeline for implementation, budgeting, infrastructure, and workforce needs for FTP is expected to be significantly lower than that of TSP because it does not involve training LLMs from scratch [114]. Typically, FTP uses readily available, pretrained proprietary or open-source LLM with open-source licenses for modifying the source code as per user needs. The number of open-source LLM offerings has continued to increase with time with communities, such as Hugging Face hosting leaderboards comparing their performance. Managed services by CSPs can assist in setting up multistep tasks across systems and data sources, generate knowledge bases from private data sources for FTP, and implement safeguards on inputs and outputs adhering to governance and responsible AI.

#### Risks

FTP will demand resources, access to quality data, relevant prompts (eg, input-output pairs), protected time for subject matter experts, and agile implementation strategies for adapting the pretrained LLMs for specific tasks. While automated approaches have been proposed for prompt engineering [128], prompt engineering risks for FTP may be relatively higher compared with fine-tuning on TSP because the training data of the pretrained models may not be domain-specific and can have potential biases. Implementation details of proprietary pretrained LLMs may not be readily accessible, limiting innovation and modification with evolving needs. Pricing of proprietary LLMs used by FTP may also increase with enhanced adoption across health care organizations, and their downtime may impact several dependent downstream applications in health care workflows. While several open-source LLMs are readily available from platforms, such as Hugging Face, these are primarily a result of crowdsourcing efforts and voluntary contributions posing challenges in translating them to enterprise tools. Dependency on an existing pretrained open-source or proprietary LLM can be a risk because these models are rapidly evolving and that could challenge active maintenance of legacy models. Open-source implementations traditionally do not support extensive documentation and training materials for onboarding. These in turn may demand workforce and digital capacity on-premises. This includes challenges in interfacing these tools with other systems and health care workflows. Open-source implementations may also be susceptible to vulnerabilities that may not be readily apparent; hence, they could be susceptible to security breaches, malicious content, malware, and ransomware attacks on models and downstream applications compromising patient privacy and leading to liabilities. Pretrained proprietary LLMs may have minimal flexibility, transparency, and interface options to downstream applications and dashboards in health care workflows. Because health care data can contain PHI, open-source and pretrained proprietary LLMs should be HIPAA compliant.

#### Economics of FTP

The economics of FTP (Table 4) are expected to be markedly lower compared with TSP as it does not involve the computationally intensive task of pretraining an LLM. More specifically, digital readiness and infrastructure and workforce costs are expected to be markedly lesser than those of TSP. In addition, FTP may require access to the annotated health care data that are several magnitudes less than the training data used in TSP. Unlike TSP, fine-tuning is typically accompanied by high GPU consumption for a short burst of time and ideal for availing pay-as-you-go models offered by CSPs in contrast to on-premise systems.

**Table 4:** Comparable pricing of graphics processing unit [GPU] clusters across cloud service providers (CSPs; Amazon Web Services [AWS], Azure, Google Cloud Platform [GCP], and Oracle Cloud Infrastructure [OCI]) for the fine-tuned pathway. Representative data were retrieved in June 2024.

CSP	Instance type	CPU^a^ cores	Memory (GiB)	GPUs	Per GPU memory	Total GPU memory	On demand (US $)	1 year (US $)	3 year (US $)
AWS	p3.16xlarge	64	488	8	16	128	24.48	15.91	8.39
AWS	p3dn.24xlarge	96	768	8	32	256	31.22	18.39	9.64
Azure	NC24rs_v3	24	448	4	16	64	12.24	8.98	6.52
GCP	n1-highmem-64	64	416	8	16	128	23.63	14.88	10.63
OCI	BM.GPU3.8	52	768	8	16	128	23.60	—^b^	—

^a^CPU: central processing unit.

^b^Not applicable.

#### OBP Overview

OBP includes commercial off-the-shelf LLMs typically accessed by end users through Representational State Transfer APIs (SaaS) with minimal or no local customization.

#### Benefits

Unlike TSP and FTP, OBP is usually enterprise-ready. OBPs do not require digital readiness in access to integrated datasets and data warehouses. LLMs are accessed through a web interface; hence, no budgeting needs to be allocated for the storage and computing infrastructure needs of LLMs or the workforce to support LLM implementation and maintenance, as in TSP and FTP. Because OBP is provided by multiple vendors, there is an option to choose the best-performing LLM for a given price. Transitioning between OBP services can be done with ease because there is no explicit sharing of sensitive health care data or customized LLM architecture. User training needs in OBP are minimal compared with TSP and FTP. Managed services provided by CSPs can assist OBP implementation with minimal workforce needs on-premises. This includes CSP-managed API end points that offer access to 1 or more LLMs, whose cost is proportional to the number of input and output tokens. Services provided by the 4 major CSPs in this regard include (AWS: Claude, Azure: OpenAI Service, GCP: Gemini, and OCI: Cohere). Managed services by CSPs also support OBP LLM deployment on GPUs with minimal ease and a per-hour charge (AWS: Amazon SageMaker Canvas, Azure: AI Studio, and GCP: Vertex AI).

#### Risks

Unlike TSP and FTP, OBP is completely dependent on the SaaS or infrastructure as a service option provided by the vendor, with no control over training and fine-tuning and limited transparency on potential biases and the details of the LLM model and the training data. Prompt engineering risks of OBP may be larger than those of FTP and TSP because the OBP may use models that are not domain-specific, and the absence of domain-specific knowledge may result in OBP being sensitive to prompting. Output variability can be relatively higher for OBP, leading to inconsistent results and challenging deploying these models in clinical workflows. Copyright protection may limit the extent to which OBP can reveal the implementation details, and OBP services can be black boxes. Therefore, OBP services are expected to provide generic insights that may not necessarily accommodate the digital footprints and characteristics of the population served by a health care organization. This in turn may diminish the value and utility of OBP in addressing the needs that are specific to the organization. It might not be feasible to use PHI data across OBP services that are not HIPAA compliant. Posing questions to OBP services may unintentionally compromise patient privacy, especially when the sample size of the cohort being queried is small (eg, rare disease). OBP services may pose challenges in interfacing other applications, dashboards, and workflows without the explicit involvement of the vendor. Cost is usually incurred per inference and can aggregate over time with increasing dependency and number of users. These in turn may demand user authentication, quota allocation, and auditing. The reliability of the OBP service is dependent on the vendor and not necessarily guaranteed, with the potential for pricing options to increase with increasing adoption.

#### Economics of OBP

The cost of digital readiness for OBP is expected to be minimal as OBP services are provided as ready-to-use solutions without any need for training or fine-tuning as in TSP and FTP. Ideally, OBP would not require a dedicated team to support other than training materials for end users. However, hosting open-source LLM models, such as those from Hugging Face Hub, may demand infrastructure costs on-premises or in the cloud. Alternatively, LLM models in the OBP can be availed through APIs whose charges vary based on the input and output tokens and use patterns. Use patterns need to be carefully monitored as they could gradually increase to millions of tokens per month, significantly impacting the cost of OBP long-term. Pricing estimates of LLM APIs across major CSPs are shown in Table 5 [129-132]. Due to the per-unit pricing of certain CSPs, an average of 4 characters per token is assumed in generating these estimates.

**Table 5:** Comparable pricing of large language models through application programming interface across cloud service providers (CSPs; Amazon Web Services [AWS], Azure, Google Cloud Platform [GCP], and Oracle Cloud Infrastructure [OCI]). Representative data were retrieved in June 2024.

CSP	Model	Input unit cost (US $)	Input parameters	Output unit cost (US $)	Output parameters	10,000 Input tokens (US $)	500 Output tokens (US $)	Total cost (US $)
AWS	Claude 3 Sonnet	0.003	Per 1000 tokens	0.015	Per 1000 tokens	0.030	0.008	0.038
Azure	GPT-4o global deployment	0.005	Per 1000 tokens	0.015	Per 1000 tokens	0.050	0.008	0.058
GCP	Gemini 1.5 pro	0.00125	Per 1000 characters (approximately 250 tokens)	0.00375	Per 1000 characters (approximately 250 tokens)	0.050	0.015	0.058
OCI	Cohere large	0.022	Per 10,000 transactions (approximately 2500 tokens)	0.022	Per 10,000 transactions (approximately 2500 tokens)	0.088	0.004	0.092

### LLM Applications in Health Care

**Figure 3:**
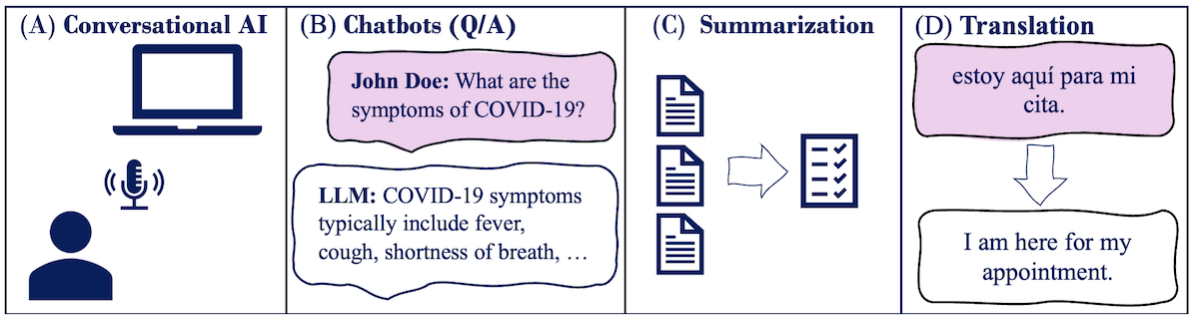
Typical health care applications of (A) large language models (LLMs) and conversational artificial intelligence (AI), (B) chatbots, (C) summarization, and (D) translation. Q/A: question and answer.

LLMs have the potential to offer health care providers new mechanisms for optimization and automation of documentation, clinical review, and direct patient communication. Its use is expected to reduce provider time in using systems, such as EHR, and improve clinical documentation while minimizing repetitive workflows. LLMs can also benefit patients by serving as an approachable tool to manage their health information assist in health literacy, appointment scheduling, and ambulatory encounters. Typical LLM applications in health care are shown in Figure 3. These applications broadly rely on the principles underlying dialogue systems [133]. They facilitate text-based and voice-based interactions between LLMs and the end user.

### Conversational AI in Clinical Practice

Conversational AI (Figure 3A) focuses on the application of AI-based approaches, such as LLMs, for developing dialogue systems [133]. It is an omnibus term that includes chatbots and virtual assistants as well as text and voice-based interaction with the end user. The ability of these systems to understand user intent and generate relevant and accurate responses has continued to improve over time [134,135]. Conversational AI-enabled systems can significantly enhance information seeking and retrieval capabilities of health care information by patient, provider, and payer. Specifically in clinical practice, conversational AI integrated within EHRs [136] can facilitate increased patient-provider engagement and reduced documentation time, minimizing physician burnout. Moreover, these ambient listening, generative AI solutions have the opportunity to dramatically reduce documentation and cognitive burden, further leading to an improved clinician EHR experience. Depending on the services needed, the integration can be pursued through TSP for a bespoke solution, FTP for modifying existing models, and the OBP pathway with minimal customization. In addition to the risks and benefits of these pathways, their deployment in EHR workflows may demand expertise in user interface design for functional integration. The focus should be on ensuring that AI-supported interactions enhance rather than impede the patient-provider relationship. Operationally, such systems streamline data management processes, leading to increased efficiency and potential financial savings due to reduced time spent on repetitive tasks. Depending on digital and analytics maturity, there could be challenges related to integration and deployment challenges and enhanced adoption. In enhancing the conversational AI interfaces within EHR systems, a more detailed exploration of specific use cases and scenarios would reveal their impact on clinical workflow, such as patient portal communications or clinical decision support. Long-term adoption studies, focusing on the acceptance and resistance among health care professionals, can provide insights into the practical aspects of implementing these technologies. Moreover, a deeper discussion on how these AI interfaces evolve through ML and adaptation to user behaviors and preferences can illustrate the potential for increasingly efficient and user-friendly medical systems. The development of guardrails on AI-moderated operations and data security, particularly in the context of sensitive EHR data, is imperative to address concerns around privacy and compliance with health care regulations.

Conversational AI economics depends on the quantity, complexity, and scale of integration. For instance, conversational AI tools for EHR demand seamless and secured integration of the tool to the EHR either directly or through third-party interfaces, training the users for enhanced adoption, and customization of the tools through fine-tuning. Training conversational AI tools is critical to maintaining high accuracy on clinical tasks and dialogue understanding impacting its adoption. Successful deployment will demand active involvement of multiple teams, including analytics, information technology, the governance team, compliance, and security. Scalability considerations are crucial if deployed across multiple facilities, necessitating robust infrastructure investments. In addition, ongoing optimization costs are incurred as the AI must continuously update to align with new medical guidelines and treatment protocols, ensuring reliability and accuracy in patient data management.

### Chatbots in Health Care

A chatbot is a conversational AI system that enables communication between computers and humans using natural language in completing specific tasks, such as question answering (Figure 3B). LLM-based chatbots are transforming the way patients, providers, and payers interact within health care settings [137]. They have the potential to improve health care outcomes by providing timely health information to patients, assisting in patient education and interventions, improving operational productivity by assisting decision-making processes, and reducing administrative tasks and overhead costs [138]. Chatbots can also assist in individualized services, including symptom assessment through virtual consults, appointment scheduling, and improving health literacy by making health care information accessible in a preferred language [139-141]. In developing chatbots, health care systems may opt for TSP for a completely customized solution, FTP for adapting existing models, or OBP for ready-to-use prebuilt templates. In addition to the risks and benefits of these pathways discussed earlier, chatbot implementations demand a good understanding of patient engagement strategies and health care communication norms. Successful deployment should ensure privacy and security of patients, adhere to regulations, and establish necessary guardrails [110]. Their design should also prioritize empathy and cultural sensitivity to ensure inclusive and respectful interactions with diverse patient populations in addition to minimizing bias and assumptions in conversations [137]. These, in turn, are expected to build trust and harmony with users, leading to enhanced adoption [142]. Successful chatbot design should also accommodate nuances of human interactions (eg, patient emotions and expectations). These in turn may demand well-articulated prompt engineering, fine-tuning, and optimization of the parameters in the underlying model [143]. Incorporating feedback from patients and health care providers will offer a deeper understanding of user experience and areas for improvement. Chatbots can also be integrated with other digital health technologies and workflows, such as telemedicine and electronic medical record. In addition, it can assist in personalization of services, tailoring interactions based on individual patient profiles and needs, significantly impacting patient engagement. Integrating chatbots with predictive analytics can also assist in assessing the usefulness of these tools by incorporating feedback.

The economics of LLM-powered chatbots in health care can be impacted by integration, compliance needs, feature complexity, and deployment scale. OBP is generally the least expensive, potentially costing a few thousand to tens of thousands of dollars annually, based on a subscription model. However, these figures can easily grow with the frequency of use and number of users. Fine-tuning existing models can cost tens to hundreds of thousands of dollars, depending on the extent of customization and licensing fees. Developing a highly customized chatbot from scratch is the most expensive option, with expenses running into hundreds of thousands to millions of dollars. Additional costs arise from ongoing maintenance, server costs, updates, and compliance with regulations such as HIPAA or GDPR, which necessitate robust security measures. The complexity of features, such as multilingual support advanced diagnostic capabilities, and the scale of deployment can also significantly impact the overall cost.

### Summarization in Health Care

LLMs excel in summarization (Figure 3C), tasks critical for managing extensive medical documentation and improving clinical workflows [144]. These AI-driven systems can distill complex medical records into concise summaries for improved decision-making and patient management. Summarization also reduces task load while improving documentation quality, operational efficiency in processing documents, and financial savings by reducing the time and resources spent on administrative tasks. Summarization can also succinctly capture details of patient-provider conversations in team settings comprising a large number of clinicians, resulting in enhanced care continuity, coordination, and overall quality. While similar in integration complexity to conversational AI systems, the implementation of LLMs for summarization specifically requires tuning the models to capture critical medical insights accurately. Summarization leverages advanced natural language understanding capabilities, a step beyond general chatbot applications to ensure that summaries are not only succinct but also clinically relevant. These can be developed through TSP for high specificity, FTP for a balanced approach, or OBP for broader applications. The summarization process requires an augmented framework comprising a group of experts in the domain of AI, clinical knowledge and medical terminology, and data-processing infrastructure for critical validation. Successful implementation of LLM summarization is expected to ensure integrity of medical information, preventing any loss of critical details in the summarization process, minimizing the risk of misinterpretation of condensed information, and seamless integration of these tools into existing clinical workflows.

The economics of summarization while overlapping with those of broader AI integrations, such as conversational AI tools, are particularly influenced by the need for high-quality training data and the development of interfaces that clinicians can use effectively within existing digital health frameworks and workflows. The cost and investment may increase for summarization tools that meet high standards of accuracy and reliability in medical contexts while minimizing risks. This includes rigorous testing and validation in real-world settings to adhere to the data handling and privacy regulations characteristic of the health care institution and industry. In addition, the ongoing maintenance to update the models with new medical information and guidelines further adds to the overall expenditure. These factors combined make the economics of summarization technologies substantial yet crucial for enhancing efficiency and decision-making in health care environments.

### Machine Translation in Health Care

Machine translation (Figure 3D) serves diverse linguistic communities by the translation of text or speech from one language to another. Its role is especially helpful in overcoming language barriers in medical communication and documentation, especially across health care organizations that serve non–English-speaking communities. Machine translation can improve patient–health care provider communication, patient understanding of instructions, and discharge summaries, as well as operational benefits by facilitating multilingual documentation and financial advantages by potentially reducing the workload for human interpreters in low-resource settings [145]. It also has the potential to assist in the transmission of critical medical information in a culturally sensitive and empathetic manner, with the potential to minimize adverse events and impact health care outcomes favorable, especially across non–English-speaking communities. Machine translation can be developed through TSP for precise, context-specific translations using data from communities served by a specific health care organization, FTP for adapting existing models to medical language nuances, or OBP for immediate implementation with off-the-shelf translation tools. In addition to the applications mentioned here, machine translation requires collaboration with linguists and cultural sensitivity advisers to ensure translations are accurate and culturally appropriate. Ethical and regulatory considerations revolve around the accuracy and cultural appropriateness of translations. There is a strong emphasis on avoiding miscommunication in critical medical contexts while respecting linguistic diversity. Challenges include the risk of mistranslation, cultural insensitivity, and loss of nuanced medical context. The impact on target populations is usually diverse. It enables payers to offer multilingual services efficiently, aids providers in delivering equitable care to non–English-speaking patients and empowers patients by providing access to medical information in their preferred native languages. Here, underlining cultural competency alongside language translation is crucial. This includes not only translating text but also understanding and conveying cultural nuances, which is critical in medical contexts with potential favorable impact outcomes. Establishing specific metrics or standards to gauge the accuracy and reliability of translations can provide a benchmark for evaluating these tools. Discussing the legal implications and responsibilities in cases of mistranslation or miscommunication is also vital to understanding the potential liabilities involved. Extensive testing may be required before deployment in high-stakes areas, such as emergency medicine, where quick and accurate translation is vital.

The economics of machine translation in health care settings largely overlap with those of conversational AI technologies, as discussed previously. Costs can vary based on the development pathway chosen: OBP solutions may offer a lower upfront cost with general translation tools available for immediate use, while the FTP and the TSP require more substantial investments to adapt or develop models that handle medical language nuances and specific community dialects. However, OBP solutions could have severe limitations in high-stakes applications compared with FTP and TSP. Costs usually include customization, system integration with existing health care IT infrastructures such as EHRs, and ongoing expenses for maintenance and updates. Additional significant expenses are incurred in ensuring accuracy and cultural appropriateness, which involves collaboration with linguists and cultural experts. This collaboration is essential to mitigate risks of mistranslation and to comply with health care communication standards. Therefore, while the base technology may be like those used in conversational AI, the specificity and critical nature of medical translations can lead to higher costs, particularly when ensuring the system meets the stringent requirements of medical accuracy and regulatory compliance.

### Conclusions

LLMs have the potential to meaningfully impact health care delivery and health outcomes. However, LLM implementations are impacted by the needs and affordability. This perspective provided 3 LLM implementation pathways (TSP, FTP, and OBP) and a road map for onboarding, enhanced democratization, and equitable adoption by the health care ecosystem. The economics, risks, and benefits of these pathways were also presented across 4 major CSPs (AWS, GCP, Azure, and OCI) to assist in choosing the best pathway for an organization. As LLMs continue to evolve [146], additional onboarding pathways are expected to join the repertoire.

The critical role of cloud-computing frameworks to support onboarding efforts from scalability, privacy, workforce, and economic standpoints was discussed. Pay-as-you-go models offered by CSPs alleviate the need for significant upfront investments while providing the ability to experiment with different pathways with the flexibility to scale and transition between pathways based on the usefulness of these tools in impacting outcomes and KPIs and use with evolving needs of health care organizations. Managed services provided by CSPs can assist in optimal management of resources and infrastructure while streamlining workflows and minimizing the need for considerable expertise across a variety of areas. These aspects are especially suited for organizations that do not have sufficient resources and upfront investment for LLM implementation. CSPs also provide privacy and security features, including confidential computing and TEEs for safeguarding sensitive health care data and maintaining regulatory compliance. The size of health care datasets used to train LLMs is often small compared with datasets used to train general-purpose models. CSPs can facilitate FL in conjunction with TEEs and deidentification in overcoming sample size constraints by enabling collaborative training strategies across health care organizations without explicit data sharing and ensuring privacy. FL approaches can result in models with enhanced generalization ability in contrast to those trained using data from a single health care organization. Because LLMs may have the potential to memorize sensitive information from training data, hindering the sharing of checkpointed models due to privacy concerns. Techniques such as differential privacy and secure multiparty computation in CSPs can mitigate such risks. CSPs also provide access to specialized hardware accelerators, presenting an opportunity to improve the efficiency and cost-effectiveness of LLM training and inference. However, it is important to consider compatibility and performance trade-offs when integrating these accelerators into existing workflows.

As LLMs are used across health care, it is crucial to consider potential challenges and unintended consequences. Choice of an LLM implementation pathway can be significantly impacted by digital readiness, infrastructure, workforce, and ethical and regulatory landscape. Overreliance on LLMs could also diminish the critical thinking skills of health care professionals. As with all AI and ML tools, optimization is an essential ingredient of LLMs. Therefore, it is essential that LLMs be used within an augmented framework to support human decision-making rather than serving as a replacement. Establishing guardrails, ethical guidelines, and training programs for ensuring the responsible use of LLMs in clinical settings is important. Providing training and support for health care professionals and actively engaging them in the LLM implementation is critical for their successful deployment and long-term adoption. Workforce is a critical ingredient for LLM implementation, deployment, and maintenance. Prioritizing the inclusion of belonging, diversity, equity, and inclusion leaders as a part of LLM development is crucial to ensuring implementation that is inclusive and representative of diverse populations. Engaging policy makers and educating them about LLM limitations and adoption in health care is critical for realistic expectations from these tools and developing the necessary regulatory frameworks. Incentives could be introduced to encourage LLM adoption across health care organizations, and KPIs should be identified to assess its impact on health care outcomes. Short-term incentives can facilitate initial adoption of a particular onboarding pathway, while long-term incentives may assist in shifting across these pathways.

Identifying onboarding pathways for LLM implementation leveraging cloud computing along with metrics to demonstrate value while incorporating the necessary regulations and guardrails of responsible AI is critical for its equitable distribution and enhanced adoption in the health care ecosystem. Widespread adoption is also expected to facilitate feedback from diverse communities served by the health care ecosystem, improving patient outcomes and operational efficiency and addressing the unique challenges and considerations in health care.

## Abbreviations

AI: artificial intelligence
API: application programming interface
AWS: Amazon Web Services
CCE: confidential computing environment
CFL: confidential federated learning
CSP: cloud service provider
DL: deep learning
DNN: deep neural network
EDW: enterprise data warehouse
EHR: electronic health record
ETL: extract, transform, and load
FL: federated learning
FTP: fine-tuned pathway
GCP: Google Cloud Platform
GDPR: General Data Protection Regulation
GPU: graphics processing unit
HIPAA: Health Insurance Portability and Accountability Act
KMS: Key Management Service
KPI: key performance indicator
LGBTQ: lesbian, gay, bisexual, transgender, and queer
LLI: Lucian Leape Institute
LLM: large language model
ML: machine learning
NLP: natural language processing
OBP: out-of-the-box pathway
OCI: Oracle Cloud Infrastructure
PHI: protected health information
SaaS: software as a service
TEE: trusted execution environment
TPU: tensor processing unit
TSP: training from scratch pathway
VM: virtual machine
